# Phase Transition toward a Thermodynamically Less Stable Phase: Cross-Nucleation due to Thin Film Growth of a Benzothieno-benzothiophene Derivative

**DOI:** 10.1021/acs.jpcc.1c06610

**Published:** 2021-12-20

**Authors:** Sebastian Hofer, Andreas Hofer, Josef Simbrunner, Michael Ramsey, Martin Sterrer, Alessandro Sanzone, Luca Beverina, Yves Geerts, Roland Resel

**Affiliations:** † Institute of Solid State Physics, Graz University of Technology, 8010 Graz, Austria; ‡ Division of Neuroradiology, Vascular and Interventional Radiology, Medical University Graz, 8010 Graz, Austria; § Institute of Physics, Karl-Franzens University Graz, 8010 Graz, Austria; ∥ Department of Materials Science, University of Milano-Bicocca, 20126 Milano, Italy; ⊥ Laboratoire de Chimie des Polymères, Faculté des Sciences, Université Libre de Bruxelles, Boulevard du Triomphe, CP 206/01, 1050 Bruxelles, Belgium; # International Solvay Institutes for Physics and Chemistry, Université Libre de Bruxelles, Boulevard du Triomphe, CP 231, 1050 Bruxelles, Belgium

## Abstract

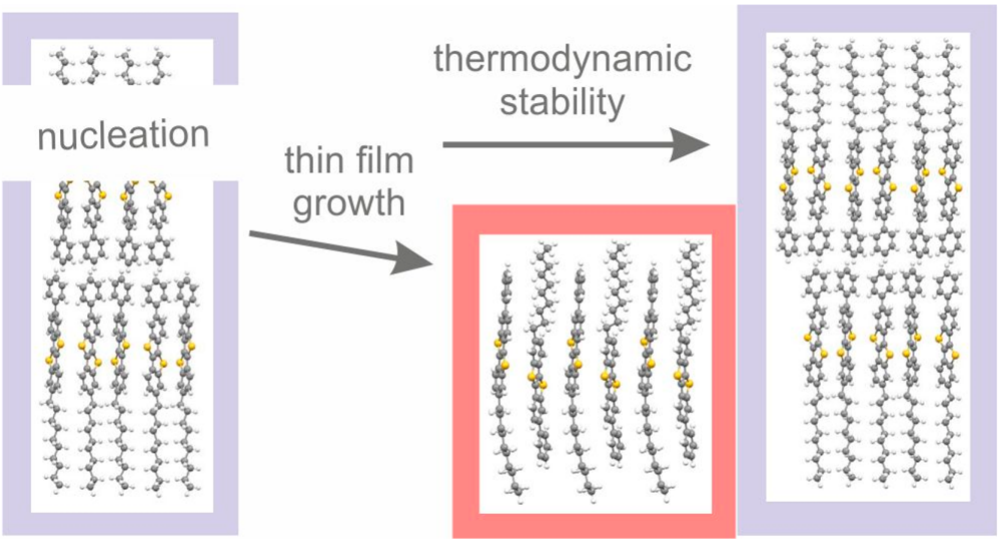

The molecule 2-decyl-7-phenyl-[1]benzothieno[3,2-*b*][1]benzothiophene is an organic semiconductor, with outstanding properties in terms of molecular packing and its use in organic electronics. The asymmetric shape of the molecule causes a double layer crystal structure at room temperature. In this work we report its thin film growth by physical vapor deposition starting from the monolayer regime up to thick films. The films are studied in terms of their morphology, crystallographic properties, and thermal stability by atomic force microscopy and X-ray diffraction methods. It is found that the bulk molecular packing of the bilayer is formed at the initial thin film growth stage. After a thickness of one double layer, a transition into a new polymorph is observed which is of metastable character. The new phase represents a single layer phase; the crystal structure could be solved by a combination of X-ray diffraction and molecular dynamics simulations. The observed thin film growth is outstanding in terms of surface crystallization: the formation of a metastable phase is not associated with the initial thin film growth, since the first growth stage represents rather the bulk crystal structure of this molecule. Its formation is associated with cross-nucleation of one polymorph by another, which explains why a metastable phase can be formed on top of a thermodynamically more stable phase.

## Introduction

Polymorphism in molecular crystals has become an important issue, since application-relevant properties depend highly on the type of phase.^1,2^ The recent efforts of defined crystallization within thin films reveals an additional source of polymorph phases due to the presence of a substrate surface during the crystallization process.^3,4^ Polymorphism with strong variation of molecular packing is possible; an important issue is the thermodynamic stability of the polymorph phases.^5,6^ Thin film metastable phases appear for most of the well-studied organic electronic molecules such as oligoacenes, oligothiophenes, or benzothieno-benzothiophene based derivatives.^7−10^

Metastable phases appear as a consequence of the growth kinetics: fast solidification processes together with weak nondirected interactions can cause changes in the molecular packing and improvable intermolecular arrangements. Restricted molecular conformation are possible.^11,12^ Frequently, metastable phases appear as a result of a thin film deposition process.^3^ Depending on the preparation method, the crystallization process can be close to or far from the thermodynamic equilibrium. On the one hand, solution processing by drop casting leads rather to the equilibrium crystal structure if the solvent evaporation is slow. On the other hand, solution processing by quick solvent evaporation or even physical vapor deposition can result in crystalline phases far from the thermodynamic equilibrium.^6^ The presence of a surface during the crystallization process plays an additional role, since the interplay in the intermolecular interaction and the molecule/substrate interaction are important parameters which determine the preferred crystallization relative to the substrate surface.^13,14^ The confinement of the molecular packing with the substrate surface can be the origin of specific polymorphs which are assigned to substrate-induced polymorphism.^15,16^

The crystallization process starts at the substrate surface. The molecular packing motifs within the initial crystal nuclei are a result of constraints determined by the substrate surface. The formed crystals do not necessarily induce a stable crystal structure for the entire film.^8,17^ Generally, it is expected that a transition to the equilibrium bulk structure may take place for crystals sufficiently decoupled from the substrate surface.^18^ However, metastable phases can be found also for thin films (e.g., of pentacene) with a nominal thickness of several hundred nanometers.^19^

The present work represents a unique observation in that context. The known equilibrium bulk phase of the performing molecular semiconductor 2-decyl-7-phenyl[1]benzothieno[3,2-*b*][1]benzothiophene (C_30_H_32_S_2_, abbreviated Ph-BTBT-10) is formed directly at the substrate surface, and subsequently a new polymorph is formed at a later growth stage. This unprecedented effect of surface crystallization has implications for the polymorphism of organic compounds at substrate surfaces.

### The Molecule Ph-BTBT-10

**Figure 1:**
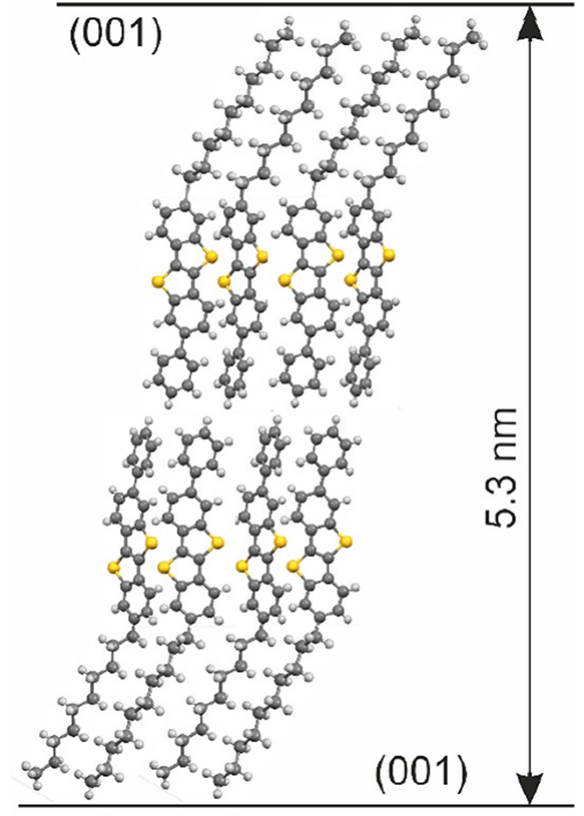
Packing of the molecules as a double layer structure present within the known bulk phase of the molecule Ph-BTBT-10. The crystallographic (001) planes are drawn, and their interplanar distance is given.

The molecular semiconductor Ph-BTBT-10 is the focus of considerable attention as it shows excellent performance in thin film transistors.^20,21^ The molecule is composed from a benzothieno-benzothiophene (BTBT) core with a phenyl ring at one terminal end of the BTBT core and with a decyl chain at the other terminal end. The molecule crystallizes in a layered structure with nanosegregation of the conjugated parts of the molecule from the decyl part.^22^ The conjugated parts pack in a herringbone pattern, typical for rodlike conjugated molecular units.^23^ Double layers are formed where two herringbone layers as well as two decyl layers are placed next to each other. The thickness of the double layer corresponds to the crystallographic (001) plane with an interplanar distance of 5.3 nm. The packing of the molecules within the bulk phase is depicted in Figure 1. The asymmetric nature of the molecule, which is a composition of a rigid part and of a flexible part, favors liquid crystalline states; the associated phase transitions are under discussion.^24,25^

## Experimental Section

The molecule Ph-BTBT-10 was synthesized according to the recently published strategy.^26^ The as-synthesized powder was purified via sublimation before use. Films with different thicknesses were deposited by physical vapor deposition onto 1 cm × 1 cm silicon substrates covered with a 150 nm thick layer of thermally grown silicon oxide. Substrates were chemically cleaned by isopropanol and acetone obtaining a surface energy of 49 mN/m with a polar part of 24 mN/m and a dispersive part of 25 mN/m (details are given in the Supporting Information and Figure S1). The substrates were inserted into a vacuum chamber, and the molecule Ph-BTBT-10 was deposited from a Knudsen cell in a vacuum with a base pressure of about 2 × 10^–8^ mbar. Films with nominal thicknesses starting from submonolayer coverages (1.5 nm) to a complete coverage of the substrate surface (6 nm) up to multilayer films with thicknesses of up to 80 nm were deposited. The nominal film thickness was determined during the deposition process with a quartz microbalance; the deposition rate was in the range of 1 nm/min.

The thin film morphology was investigated via atomic force microscopy (AFM). A Nanosurf Easycan 2 was used equipped with PPP-NCLR-50 silicon tips from Nanosensors. The investigations were performed in tapping mode, and height images as well as phase contrast images were taken. For AFM image analysis, the software Gwyddion was used.^27^

X-ray reflectivity (XRR) was carried out with a PANalytical Empyrean reflectometer in θ–θ geometry using Cu Kα radiation. At the incident beam side, a parallel beam X-ray mirror was used for monochromatizing. At the diffracted beam side an antiscatter slit and a 0.02 rad Soller slit were used together with a PIXcel3D detector operating as a point detector. Temperature dependent measurements were performed with a DHS 900 heating stage from Anton Paar Ltd. Graz.^28^ The experiments were performed under a nitrogen atmosphere. The data were converted into reciprocal space by the scattering vector *q*_*z*_ along the *z*-direction (perpendicular to the substrate surface) with
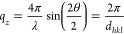
using λ as the wavelength of the primary X-ray beam, 2θ as the scattering angle, and *d*_*hkl*_ as the interplanar distance of the (*hkl*) plane. XRR data of thin films (nominal thickness ≤12 nm) were fitted with the software STOCHFIT to obtain the electron density distribution along the *z*-direction (perpendicular to the substrate surface) by a free model approach.^29^ The results are scaled to the electron density of the amorphous silicon oxide layer, which results in absolute values of the electron density of the organic layer.^30^ The X-ray diffraction peaks of thick films (nominal thickness ≥20 nm) were evaluated in terms of vertical crystal size by fitting the peak width and the associated Laue fringes.

Grazing incidence X-ray diffraction (GIXD) was carried out at the beamline XRD1 at Elettra Sincrotrone Trieste with a wavelength of 1.4 Å using for the primary X-ray beam an incidence angle of α_i_ = 0.8° on a goniometer in kappa geometry.^31^ A PILATUS 2 M detector was used to collect the diffracted intensity. To improve statistics, the sample was rotated during the measurement and the diffracted intensity was integrated over an exposure time of 30 s for a sample rotation of 60°. Data from GIXD are presented as a function of the scattering vector *q*. The components of the scattering vector are determined for each detector pixel from the incident angle α_i_ and from the outgoing angle α_f_ in the sample coordinate system together with a calibration measurement on a LaB_6_ film. Finally, reciprocal space maps are drawn as a function of *q*_*z*_ (component chosen perpendicular to the substrate surface) and of *q*_*xy*_ (component chosen parallel to the substrate surface). The data were evaluated with the use of the in-house developed software package GIDVis.^32^ The resulting reciprocal space maps are corrected on the basis of geometrical correction factors, i.e., Lorentz and polarization factors.

Determination of the molecular packing within the polymorph phase was performed by an experimental/computational approach. In a first step the lattice constants were determined by indexing of the GIXD pattern using a recently developed indexing routine.^33^ The crystallographic unit cell was used as input for a molecular dynamics (MD) simulation for a determination of the molecular packing. These simulations were carried out with the LAMMPS software package^34^ using the CHARMM general force field version 3.0.1.^35^ Several thousand trial structures are generated by placing randomly oriented molecules in an expanded unit cell (140%). During the simulation run the starting configuration was relaxed and reduced to the experimentally determined unit cell size. Resulting structures are clustered on the basis of their packing motifs and their energies. Final assignment of the obtained molecular packing to a crystallographic structure was performed on the basis of a comparison of the calculated structure factors of the Bragg peaks with the experimental intensities from the GIXD measurements.

## Results

The work presents a combined experimental approach to characterize the crystallization within the initial stage of thin film growth (the nucleation process) and relate the results to the development of crystallization at subsequent growth stages. Thin film morphologies are characterized by a combination of microscopic methods with integral X-ray scattering techniques, and the results are related to crystallographic properties obtained by X-ray diffraction techniques. Molecular dynamics simulations complete the picture of the thin film growth scenario.

**Figure 2:**
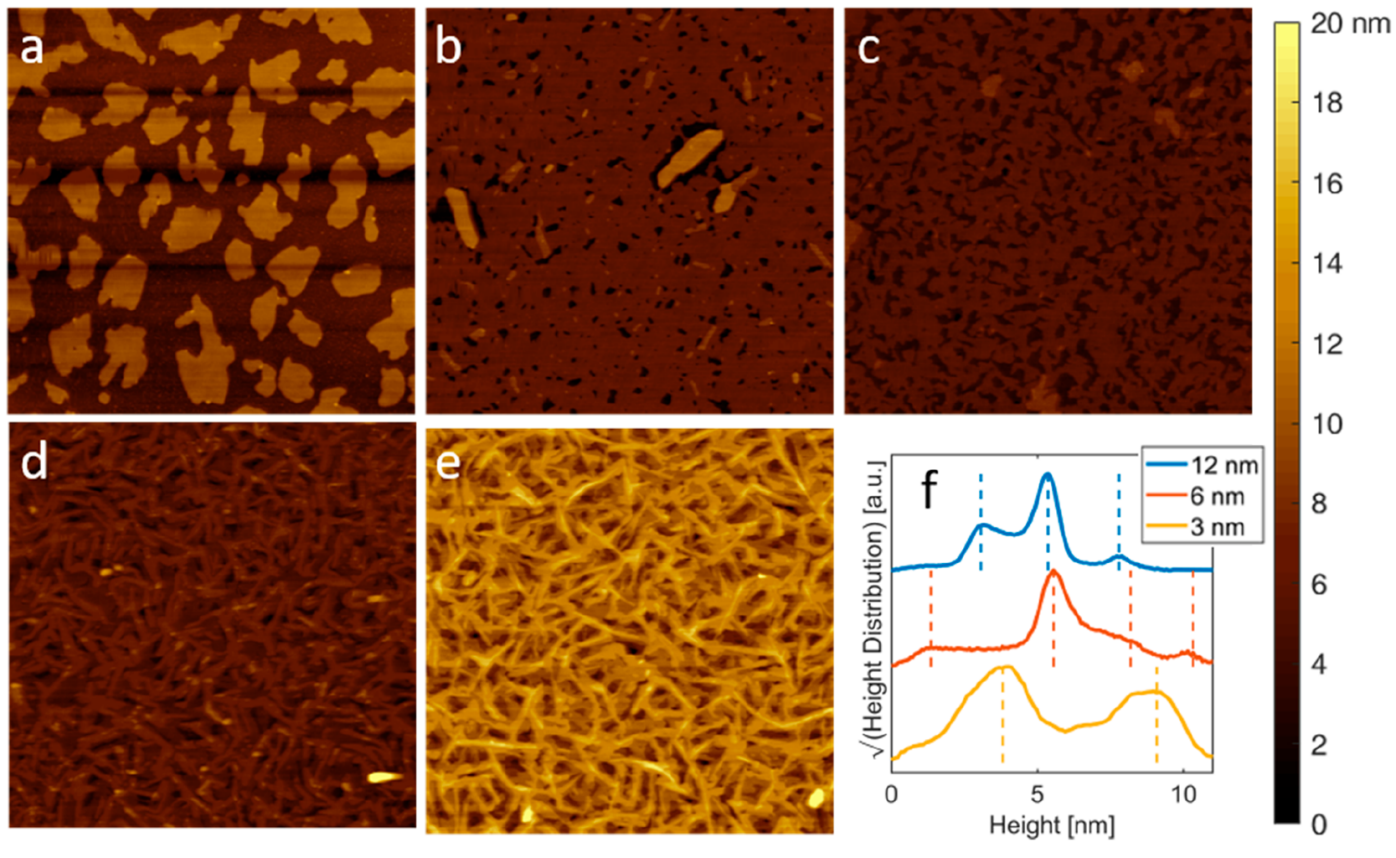
Atomic force micrographs (scan size 10 μm × 10 μm) of thin films of the molecule Ph-BTBT-10 deposited by physical vapor deposition with nominal film thicknesses of 3.0 (a), 6.0 (b), 12.0 (c), 20 (d), and 60 nm (e) as well as height distributions for films with thicknesses of 3, 6, and 12 nm with dashed lines indicating step heights of 5.2 or 2.5 nm (f).

In a first step the morphology of the films was investigated by atomic force microscopy. The corresponding AFM micrographs are depicted in Figure 2. Characteristic morphologies of submonolayer films with a nominal coverage of 3.0 nm, to a complete coverage of the substrate surface (6 nm), up to thick films with a thickness of 60 nm, are shown.

The first growth morphology is individual islands with a characteristic lateral size in a range of about 1 μm (Figure 2a). The characteristic height of these islands is determined by line scans, and a value of 5.2 nm is found (Figure S2). This island height reveals that a double layer structure is present within the initial growth stage (compare Figure 1). The height distribution function reveals an average height of the islands at 5.2 nm and a coverage of the substrate surface of about 42% (Figure 2f). This result of the AFM study agrees with the nominal film thickness determined by the quartz microbalance during the deposition process.

With increasing film thickness, the islands coalesce and films with a closed layer appear (Figure 2b). However, the film is not completely homogeneous due to the presence of open pores. Additionally, bimodal growth is found on top of the closed layer by the appearance of terraced islands. The typical height differences are visible in the height distribution function (Figure 2f). A height of 4.2 nm between the closed layer and the substrate surface (depth of the pores) is found, and characteristic step heights of 2.4 and 4.7 nm are observed between the closed layer and islands with two different height levels. The typical heights are additionally shown by a selected line scan (Figure S2).

At a nominal film thickness of 12 nm a layered structure is observed (Figure 2c). Three layers are identified. The height distribution function reveals layers with a height difference of 2.5 nm (Figure 2f, Figure S2). On further deposition of thicker films, the morphology changes significantly: at a thickness of 20 nm, elongated structures appear with ridgelike character (Figure 2d). This morphology is more pronounced at larger film thicknesses (60 nm) with highly branched ridges (Figure 2e). No further change in the morphology is observed for films up to a thickness of 80 nm.

The characteristic heights observed in the AFM studies can be compared with unit cell dimensions of a known crystallographic structure. The island height of the first growth stage with 5.2 nm is close to the interplanar distance of the (001) plane (*d*_001_ = 5.304 nm). We conclude that the initial growth stage represents a double layer structure as it is the case for the known crystallographic phase (compare Figure 1). However, the change of the growth stage at a film thickness of between 6 and 12 nm is accompanied by a terrace height of 2.5 nm. This represents rather a single layer structure. In both cases the molecules are aligned with their long molecular axes perpendicular to the layer, i.e., perpendicular to the substrate surface. Please note that the variation of the layer thickness depends on the exact tilt angle of the molecules within the layer as well as on the conformation of the molecules, i.e., the angle between the aromatic part and the alkyl part of the molecule.

**Figure 3:**
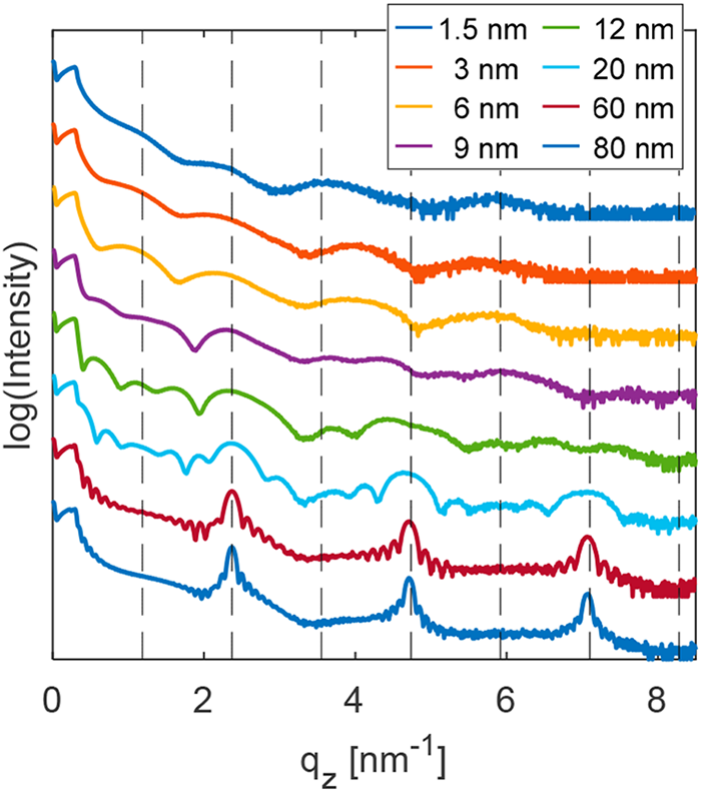
Specular X-ray reflectivity curves of thin film with varying nominal thickness. Vertical dotted lines indicate peak positions of the 00*l* diffraction peaks calculated on the basis of the known crystallographic bulk of Ph-BTBT-10.

Area integrated information about the thin film morphology together with crystallographic information is obtained by X-ray reflectivity. The results on a sample series starting with a nominal thickness of 3 nm up to thick films with a thickness of 80 nm are depicted in Figure 3. In all cases Kiessig fringes are clearly visible, revealing the presence of homogeneous layers: samples with higher coverages (larger than 20 nm) show additional Bragg peaks revealing the crystallographic order in the deposited films.

**Figure 4:**
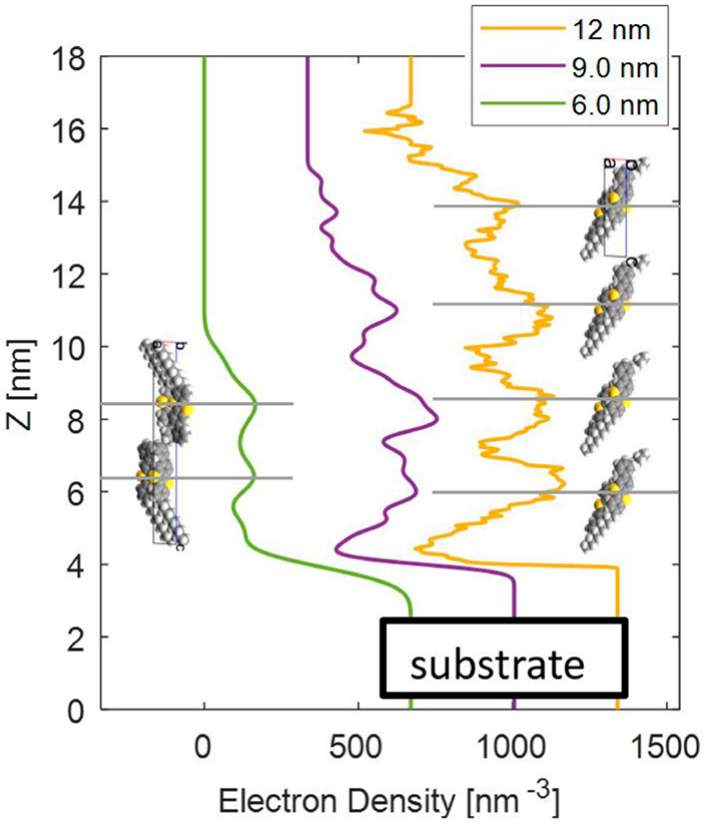
The *z*-dependence (perpendicular to the substrate) of the electron density of three selected X-ray reflectivity curves with nominal thicknesses of 6, 9, and 12 nm. The curves are shifted for clarity. The electron density of the substrate is set to the value of 670 nm^–3^ for SiO_2_.^38^ The molecules are drawn in scale with their respective orientations relative to the substrate; regions with enhanced electron densities are marked by vertical gray lines.

In a first step the initial thin film formation is discussed. Films with a nominal thickness of 1.5, 3, 5, and 6 nm are investigated and fitted in terms of layer thickness and average mass densities (Table S1). Thicknesses between 5.4 and 5.6 nm are obtained, which reveal that a double layer structure is formed at the substrate surface. The electron density distribution is calculated for the film with a closed double layer, and the corresponding fit is shown in Figure S3. The electron density distribution along the *z*-direction of the 6 nm film (Figure 4) reveals the internal structure of the double layer. The differences in the total electron densities of the decyl side chains and of the conjugated parts of the molecule make a determination possible.^36^ It is found that the two aromatic parts of the molecule point toward each other and the outer regions of the double layer are formed by the decyl chains.

In a next step the X-ray reflectivity curves of films with intermediate film thicknesses are considered. The electron density distributions cannot be explained by a double layer arrangement of the molecules; rather, the variation of the electron density follows a single layer structure (Figure 4). Single layers are found for the 9 nm film and for the 12 nm film, and the distances between the single layers varies from 2.4 to 2.7 nm. A repeating distance of 2.65 nm is plotted in Figure 4 by gray lines. The low number of repeating planes does not allow observation of the defined stacking of single layers by a Bragg peak. The superposition of Bragg diffraction and Kiessig fringes from X-ray reflectivity does not allow a clear assignment of an observed intensity maximum to a defined interplanar distance.^37^ However, starting at a film thickness of 20 nm, defined Bragg peaks appear at *q*_*z*_ = 2.37 nm^–1^, together with higher order reflections at *q*_*z*_ = 4.71 nm^–1^ and *q*_*z*_ = 7.07 nm^–1^ arising from crystallographic net planes with an interplanar distance of 2.64 nm.

The presence of diffraction peaks reveals a crystalline state of the molecule Ph-BTBT-10. The width of the Bragg peaks reveals the *z*-height (perpendicular to the substrate surface) of the crystallites. The height of the crystallites is in good agreement with the nominal film thickness; the values are given in Table S1. Defined Laue fringes are observed around the Bragg peaks, revealing the homogeneity of the crystal height; the defined Kiessig fringes at low *q*_*z*_ values (0.3–1 nm^–1^) reveal the homogeneity of the overall film.

The observed peak positions cannot be explained by the known bulk phase of the molecule Ph-BTBT-10. The expected 00*l* peak positions of the bulk phase are shown by vertical dashed lines in Figure 3 arising from an interplanar distance of 5.30 nm. We observe a crystal structure with an interplanar distance of 2.64 nm. In contrast to the double layer structure of Ph-BTBT-10 present in the known bulk phase, a new phase is found which represents a crystallographic structure composed of single layers. This phase is denoted in the following text as a “thin-film phase”.

**Figure 5:**
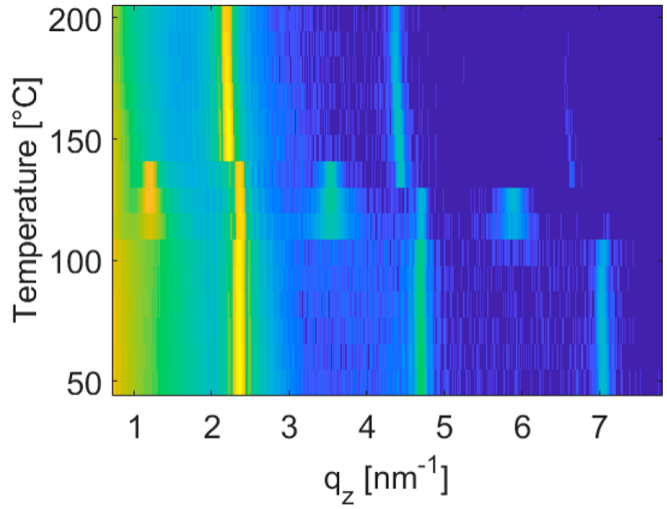
In situ temperature dependent X-ray reflectivity measurements of a Ph-BTBT-10 thin film with a nominal film thickness of 80 nm in a waterfall plot.

To study the thermodynamic stability of the thin-film phase, XRR investigations are performed as a function of temperature. A sample with a nominal thickness of 80 nm is heated at a rate of 1 °C/min while the diffraction signal is recorded. Figure 5 shows X-ray diffraction curves in a waterfall plot.

At low temperatures three diffraction peaks are observed which are at the characteristic peak positions of the thin-film phase representing a single layer structure (see above). At a temperature of 115 °C a phase transition happens. The appearance of a diffraction peak at *q*_*z*_ = 1.18 nm^–1^ together with higher order reflections represents the characteristic fingerprint of the double layer structure (bulk phase). Please note that the bulk phase is reported to be the thermodynamic stable phase of the molecule Ph-BTBT-10 with stability up to 143 °C.^20^ At that temperature a transition to a liquid crystalline state is confirmed. In our measurements this transition is observed at a temperature of 146 °C. Again, a single layer structure appears. On the basis of the exact peak positions, these phases can be assigned to the *crystal smectic E* phase of the molecule Ph-BTBT-10.^39^ This phase is stable in a temperature regime up to 210 °C.^20^ The diffraction patterns of the thin-film phase and the crystal smectic E phase show strong similarities, but a small and significant shift in the peak position is noticeable which reveals that separate phases of Ph-BTBT-10 are present.

**Figure 6:**
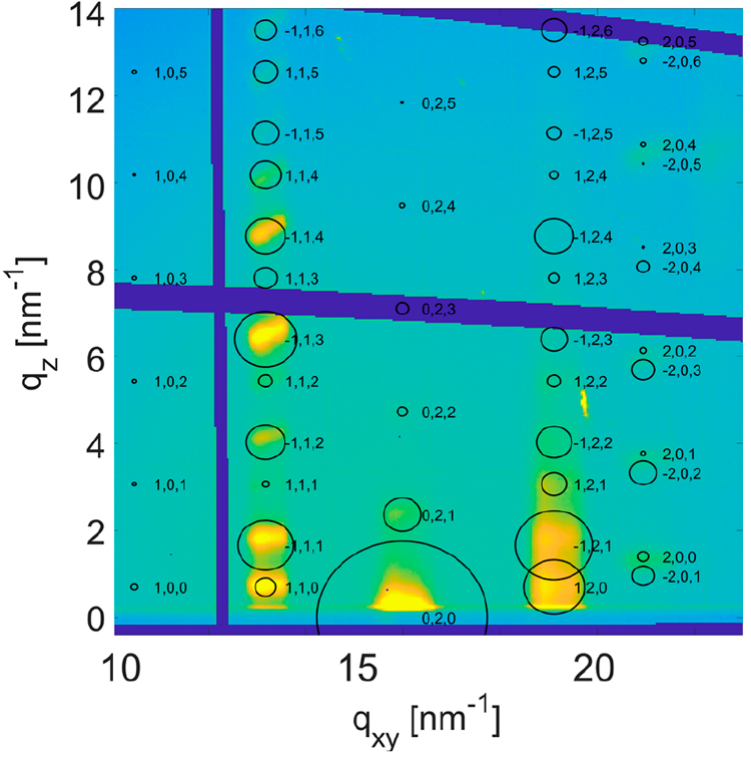
Reciprocal space maps of a thin film with a thickness of 60 nm. Intensities are plotted in logarithmic scale. Peak positions are based on the crystallographic unit cell of the thin-film phase; the areas of the circle are proportional to the structure factors of the diffraction peaks.

To study the crystallographic structure of the thin-film phase, GIXD investigations were performed. Figure 6 shows the reciprocal space map for a film with a thickness of 60 nm. A large number of diffraction peaks are visible, indicating a high degree of crystallographic order. The diffraction pattern was indexed including the Bragg peak observed in the specular diffraction experiment (Figure 3).^33^ A crystallographic unit cell with lattice constants of *a* = 0.600 nm, *b* = 0.786 nm, *c* = 2.673 nm, α = 90°, β = 93.24°, and γ = 90° was found. The calculated peak positions are given by the center of the circles within Figure 6. Assuming that the unit cell accommodates two molecules results in a mass density of 1.212 g cm^–3^. On the basis of the crystallographic unit cell, the diffraction peaks of the specular diffraction measurements could be assigned to Laue indices 00*l* (Figure 3).

The molecular packing within the crystal structure was determined by molecular dynamics simulations. The geometry of the crystallographic unit cell is used as an input parameter. The finally selected crystal structure explains the strongest intensities of our GIXD pattern reasonably well. The main diffraction peaks are along *q*_*xy*_ = 13.2, 16.0, and 19.1 nm^–1^; this arrangement is a fingerprint for herringbone packing of the aromatic units of the molecules.^8^ Additionally, the alternating peak intensities along *q*_*xy*_ (e.g., 112, −113, 113, −114) reveal that even fine details of the molecular packing are explained reasonably well. Small differences between the calculated and experimental diffraction pattern arise; they reflect the uncertainty of the experimental/theoretical approach of crystal structure solution.^40−42^

**Figure 7:**
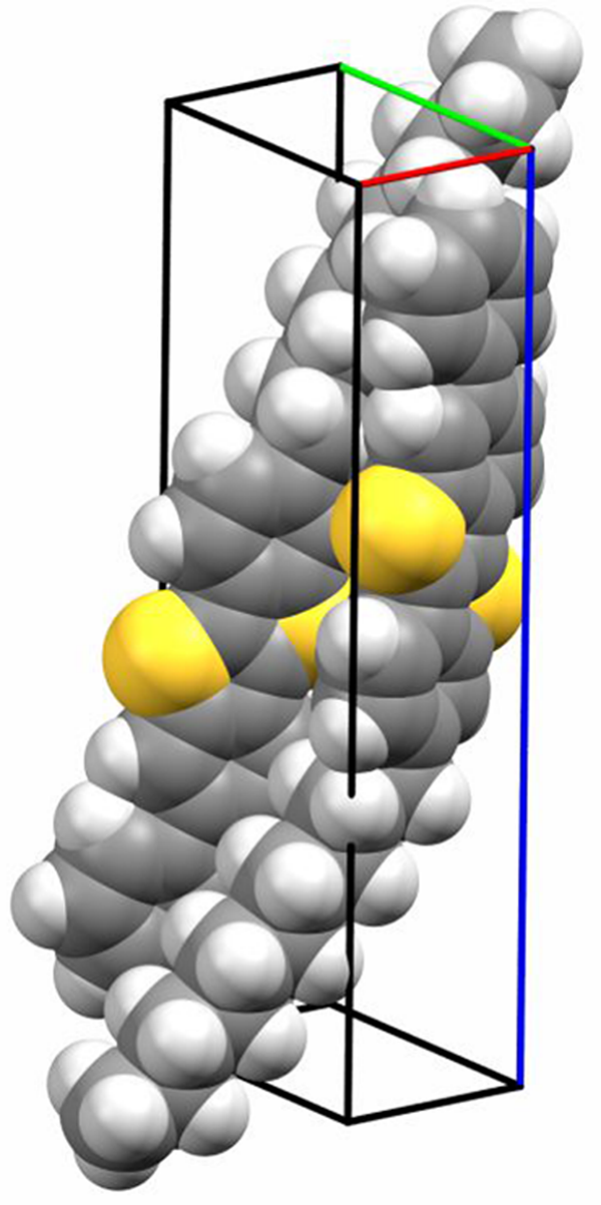
Molecular packing of Ph-BTBT-10 molecules within the thin-film phase, representing a single layer structure with antiparallel molecules.

The packing of the molecules within the crystal structure is depicted in Figure 7. We found that two molecules represent the asymmetric unit; the molecules are antiparallel to each other. The BTBT cores are in a herringbone arrangement with a herringbone angle of 34.6°. Moreover, it is important to mention that the terminal ends of the molecules (alkyl chains on one side and phenyl rings at the other side) do not form a continuous plane. This means the individual (single) layers within the crystal structure are not fully separated from each other; a minor interdigitation of neighboring layers is observed within the crystal structure of the thin-film phase.

**Figure 8:**
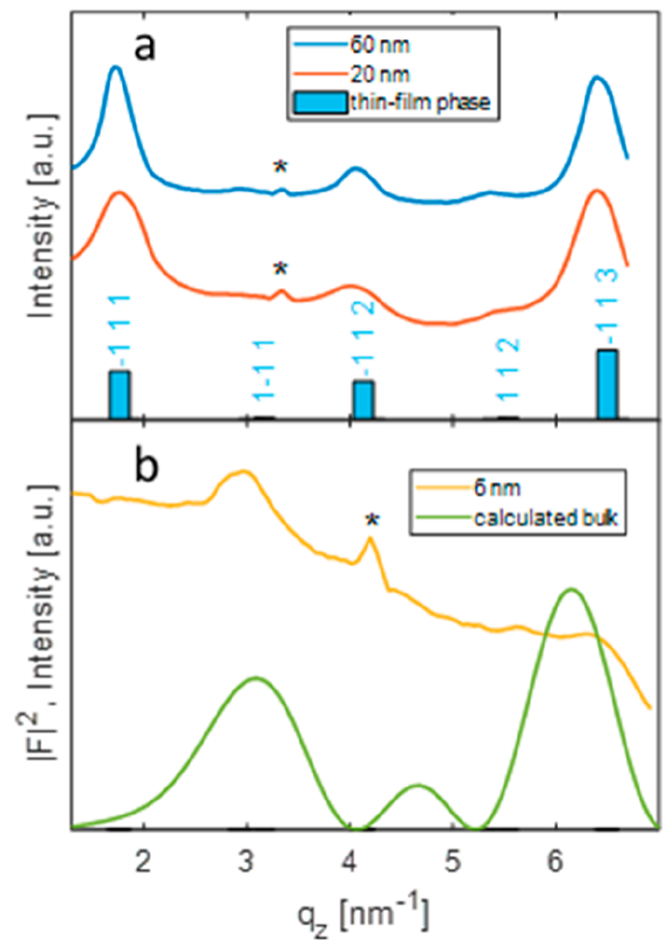
Intensity of diffraction peaks as a function of *q*_*z*_ at constant *q*_*xy*_ = 13.2 nm^–1^ for films of different thicknesses. Features marked with an asterisk (∗) arise due to enhanced intensity at detector edges. Experimental results and calculated peak pattern of the thin-film phase (a) and intensity distribution of the bilayer film in comparison with the square of the structure factor calculated on the basis of the bulk phase (b).

In a final step, we discuss the GIXD data of films as a function of film thickness. Figure 8 presents the intensity distributions as a function of *q*_*z*_ at fixed *q*_*xy*_ = 13.2 nm^–1^; a series of diffraction peaks is found along that particular direction. The experimental results of the 20 and 60 nm films are plotted, and the calculated peak pattern reveals strong diffraction peaks at 1.77, 4.16, and 6.51 nm^–1^. Good agreement is found with calculated intensities from the thin-film phase (Figure 8a). The bilayer sample (thickness 6 nm) represents a two-dimensional crystal; therefore, the measured intensities along the *q*_*z*_ direction represent the square of the structure factor.^43,44^ Enhanced intensities are found at 2.9 and 6.3 nm^–1^ which peak out from a high experimental background. (Figure 8b). We compare these peaks with the square of the structure factor at specific *q*_*z*_ positions calculated on the basis of the known bulk phase (Figure 8b, green line). The calculation reveals enhanced intensities exactly at the peak positions observed experimentally. However, a difference in peak width is observed between the experimental data and the calculation of the structure factors. An explanation can be the presence of multilayers at the initial growth stage (compare Figure 2b,f) which would narrow the experimental peak width. Nevertheless, the observation of a peak series located at *q*_*xy*_ = 13.4 nm^–1^ together with a comparable intensity distribution along *q*_*z*_ reveals that the molecular packing within the initial growth stage is comparable with the molecular packing within the double layer structure present in the bulk phase.

Interestingly, the resulting electron density fits (Figure 4) hint that a rearrangement toward a single layer structure appears when thicker films are grown. This might be because the molecules do not have time to arrange themselves into their energetically more favorable double layer bulk packing before another layer is deposited onto them, thereby stabilizing the single layer structure. Whether this really happens already in the first layer or if a fully covered double layer needs to be present to support the thin film structure formation cannot be definitely answered at this point.

## Discussion

The thin film growth of organic materials is important for a fundamental understanding of the crystallization process at surfaces, but also more broadly for the polymorphism of organic compounds, which is essential for applications. Specific morphologies as well as new polymorph phases can appear. This work presents a thin film growth study of an asymmetric molecule consisting of two segments: a conjugated part on one side and a decyl part on the other side. AFM studies reveal a double layer structure at the initial thin film formation (up to a film thickness of 6 nm) and a transition to a single layer structure at larger thicknesses. This microscopic observation could be confirmed by X-ray reflectivity and grazing incidence X-ray diffraction. It is shown that the molecular packing within the initial film growth represents the known crystal structure of the bulk phase while a new polymorph is formed at larger film thickness. The new phase—denoted as the thin-film phase—is a single layer structure with a different molecular packing in comparison to the known bulk phase. While the bulk phase represents a double layer structure with head-to-head arrangement of the molecules, the thin-film phase is a single layer structure composed by antiparallel molecules. A transition from the thin-film phase to the bulk phase is possible as a diffusionless transformation, since both phases are composed by molecules with antiparallel orientations. The observation of this transition at a temperature of 120 °C reveals that the thin-film phase is a metastable state.

Theoretical investigations of the molecular packing are based on the transitions from double layer structures (as present in the bulk phase) to single layer structures.^24,25^ Two different types of single layer structures are predicted. One of the predicted single layer structures is represented by separation of the conjugated parts from the decyl parts (so-called nanosegregation) showing strong interdigitation of the decyl chains from neighboring layers. This structure is found in the crystal smectic E phase at temperatures above 143 °C.^39^ The second predicted single layer structure is a mixed layer system with antiparallel molecules. This type of structure is found in the work presented here. All three cases—the bulk phase as well as the two nanosegregated phases—show a herringbone arrangement of the conjugated units of the molecule.

An outstanding observation is that the known bulk phase is formed at the initial growth stage; a double layer structure with a thickness of about 5.5 nm is formed. The molecules are in a head-to-head arrangement, so the conjugated parts are located at the center of the double layer and the decyl chains forming the outer regions (Figure 1). XRR as well as GIXD investigations reveal that this double layer structure shows a molecular packing known from the crystallographic structure of the bulk phase. In a subsequent step the thin-film phase appears which is in a metastable state. This observation is reversed in comparison to other known examples of thickness-induced polymorphism in organic films. Normally, metastable phases are formed at the initial growth stage directly at the substrate surface and a transition to stable bulk phases appears at later growth stages.^8,45,46^

The effect observed here—the nucleation of a new polymorph on top of another one—can be understood in relation to cross-nucleation, a phenomenon encountered in the melt growth of polymers^47,48^ but also for molecular crystals.^49,50^ New polymorphs form, since the growth velocity is faster than for the initial crystal structure, independent of their thermodynamic stability.^49,51^ Crystallization of molecules by physical vapor deposition involves different processes such as adsorption and migration of single molecules at surfaces and changes of the orientation and conformation of the molecules due to crystallization. For our situation two distinct situations are present for the orientation of the molecules: the double layer structure (located directly at the substrate surface) consists of two separated layers with either head-down or head-up orientation. The metastable thin-film phase possesses both orientations of the molecules combined in a single layer. This means that the crystallization kinetics may differ fundamentally for both types of crystal structures.

A further role may be played by confinement of the molecular packing with the substrate surface.^8,16^ In our case the bulk molecular packing of the bilayer exhibits the possibility of surface confinement, while the molecular packing within the thin-film phase requires interdigitation of the neighboring decyl layers. This means that the thin-film phase does not have densely packed edges terminating the molecular layer. This can hinder the formation of the single layer phase directly at a rigid substrate surface such as silicon oxide.

## Conclusion

Thin films of Ph-BTBT-10 were grown via physical vapor deposition onto silicon substrates. The film thickness was varied between 1.5 and 80 nm. In the regime up to 6 nm the film morphology and diffraction data indicate the growth of the well-known bulk structure of the molecule, while thicker films begin exhibiting a new polymorph phase. A new polymorph (denoted a thin-film phase) is found by indexing of GIXD patterns, which is used in a subsequent step to solve the structure with a computational approach. Although quite similar in peak positions, the bulk phase and the thin-film phase clearly differ in their peak intensities, which reflects the strong difference in molecular packing. While the bulk phase shows a double layer structure with a head-to-head arrangement of the molecules and nanosegregation of the conjugated core and the decyl chains, the thin-film phase shows a single layer system where aliphatic and aromatic residues are intertwined. It is found that the thin-film phase is stable up to a temperature of 120 °C, where a transition to the bulk phase appears. The outstanding observation of this work is that the thermodynamically more stable bulk phase represents the initial growth state of the thin film and the metastable phase is formed at a later growth stage. These results are assigned to cross-nucleation, since a change of polymorph phase appears after nucleation during the thin film growth process.

## Supporting Information Available

Line scans of the atomic force micrographs for film thicknesses 3, 6, and 12 nm; X-ray reflectivity data together with theoretical fits for films with thicknesses of 6, 9, and 12 nm; X-ray reflectivity fits and vertical crystal size of films with thicknesses of 1.5, 3, 5, 6, 9, 12, 20, 60 and 80 nm (PDF)
Crystal information file for the thin-film phase of the molecule Ph-BTBT-10 (CIF)

## Supplementary Material

jp1c06610_si_001.pdf

jp1c06610_si_002.cif

## Accession Codes

CCDC 2097887 contains the supplementary crystallographic data for this paper. These data can be obtained free of charge via www.ccdc.cam.ac.uk/data_request/cif, by email addressed to data_request@ccdc.cam.ac.uk, or by contacting The Cambridge Crystallographic Data Centre, 12 Union Road, Cambridge CB2 1EZ, U.K. (fax +44 1223 336033).
