# Supplementary material for: Phase Transition toward a Thermodynamically Less Stable Phase: Cross-Nucleation due to Thin Film Growth of a Benzothieno-benzothiophene Derivative
Source: J Phys Chem C Nanomater Interfaces. 2021 Dec 20;125(51):28039–47. doi: 10.1021/acs.jpcc.1c06610 (PMC8724801; doi:10.1021/acs.jpcc.1c06610)
Supplement: Supplementary file 1 — jp1c06610_si_001.pdf [file jp1c06610_si_001.pdf]

Supporting Information

# A Phase Transition Towards a Thermodynamically Less Stable Phase: Cross Nucleation due to Thin Film Growth of a Benzothieno-Benzothiophene Derivative

Sebastian Hofer[a], Andreas Hofer[a], Josef Simbrunner[b], Michael Ramsey[c], Martin Sterrer[c], Alessandro Sanzone[d], Luca Beverina[d], Yves Geerts[e,f], Roland Resel[a]*

[a] *Institute of Solid State Physics, Graz University of Technology, Austria*

[b] *Division of Neuroradiology, Vascular and Interventional Radiology, Medical University Graz, Austria*

[c] *Institute of Physics, Karl-Franzens University Graz, Austria*

[d] *Department of Materials Science, University of Milano-Bicocca, Milano, Italy*

[e] *Laboratoire de Chimie des Polymères, Faculté des Sciences, Université Libre de Bruxelles, Belgium*

[f] *International Solvay Institutes for Physics and Chemistry, Université Libre de Bruxelles, Boulevard du Triomphe, CP 231, 1050 Bruxelles, Belgium*

* roland.resel@tugraz.at

The surface energy of the used oxidised silicon substrates was determined by contact angle measurements. The total surface energies $\gamma_L$ of four different solvents (diiodomethane, benzyl alcohol, glycerole and water) are used together with their separated polar and dispersive parts $\gamma_L^p$ and $\gamma_L^d$, respectively. Figure S1 shows the experimental data plotted by the method by Owens and Wendt[1]; the polar and dispersive component of the surface energy was determined from the linear regression.

Figure S2 gives atomic force microscopy images of thin films in the thickness regime with nominal coverages of 3 nm, 6 nm and 12 nm, respectively. Height profiles are given along selected lines.

Figure S3 gives the respective fits of the X-ray reflectivity curves for the determination of the electron density distribution across thin films as depicted in Figure 4. The fits are performed with the software STOCHFIT[2].

Table S1 gives numerical values of layer thickness and average mass density of films with a nominal thickness in the range from 1.5 nm up to 12 nm obtained by X-ray reflectivity fits with a single layer model using the software *X'Pert Reflectivity* (PANalytical). The vertical crystal size is obtained by analysing the Laue fringes (peak broadening) for films in the thickness range from 20 nm to 80 nm.

(1) Owens, D.K.; Wendt, R.C.: Estimation of the Surface Free Energy of Polymers. *J. Appl. Polymer Sci.* **1969,** *13,* 1741–1747.
(2) Danauskas, S. M.; Li, D.; Meron, M.; Lin, B.; Lee, K. Y. C. Stochastic Fitting of Specular X-Ray Reflectivity Data Using StochFit. *J. Appl. Crystallogr.* **2008**, *41* (6), 1187–1193.

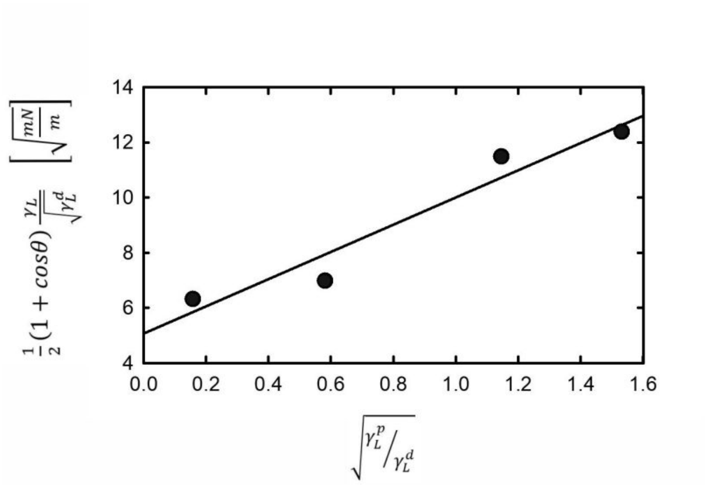


Figure S1: Contact angle θ plotted by the method of Ownes and Wendt together with a linear regression for the determination of the surface energy of the used silicon oxide surfaces.

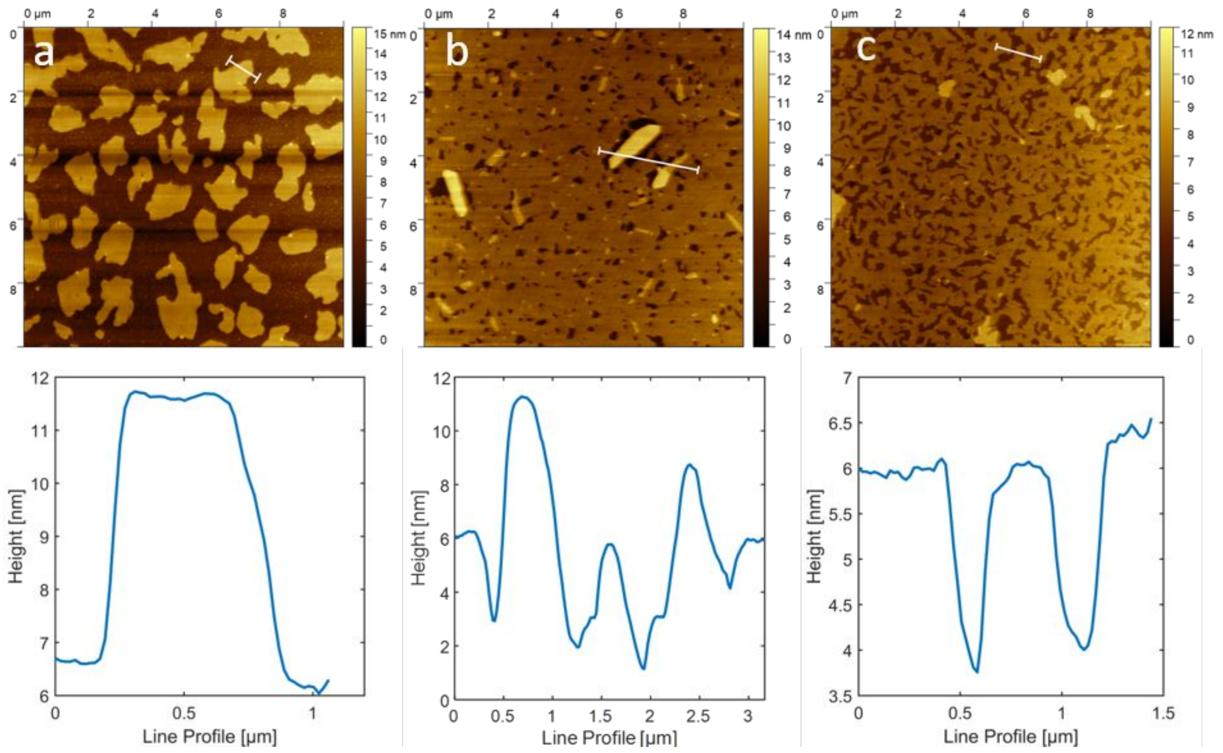


Figure S2: Atomic force microscopy micrographs with a (scan size 10 µm x 10 µm) of thin films of the molecule Ph-BTBT-10 deposited by physical vapour deposition with nominal film thicknesses of 3.0 nm (a), 6.0 nm (b) and 12.0 nm (c) with height profiles along marked lines.

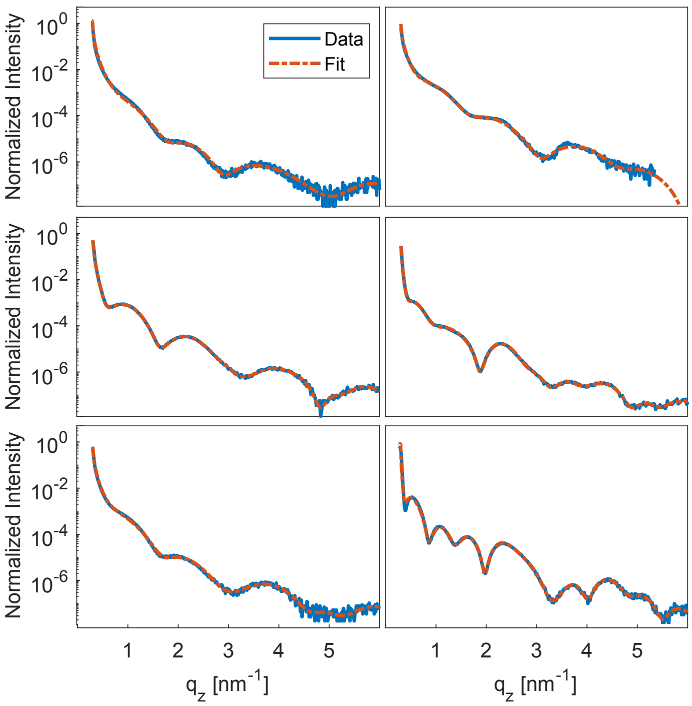


Figure S3: X-ray reflectivity data and resulting fits based on the electron density distribution by a free model approach for films with a nominal thickness of 1.5 nm, 3.0 nm, 5.0 nm, 6.0 nm, 9.0 nm and 12 nm (a-f)

| nominal thickness [nm] | thickness [nm] | mass density [g/cm$^3$] | vertical size [nm] | mean squared log-error $\frac{\|\log(y) - \log(y_{ref})\|^2}{N_{samples}}$ | Normalized root mean square log error $\frac{\|\log(y_{ref}) - \log(y)\|}{\|\log(y_{ref}) - \mathrm{mean}(\log(y_{ref}))\|}$ | Normalized mean square log error $\frac{\|\log(y_{ref}) - \log(y)\|^2}{\|\log(y_{ref}) - \mathrm{mean}(\log(y_{ref}))\|^2}$ |
|---|---|---|---|---|---|---|
| 1.5 | 5.5 | 0.36 | | 0.0887 | 0.0781 | 0.0061 |
| 3.0 | 5.7 | 0.64 | | 0.0635 | 0.0672 | 0.0045 |
| 5.0 | 5.6 | 0.79 | | 0.0188 | 0.0400 | 0.0016 |
| 6.0 | 5.6 | 0.74 | | 0.0666 | 0.0678 | 0.0046 |
| 9.0 | 8.4 | 0.80 | | 0.0081 | 0.0294 | 0.0009 |
| 12 | 11.4 | 1.06 | | 0.0275 | 0.0519 | 0.0027 |
| 20 | | | 20 | | | |
| 60 | | | 50 | | | |
| 80 | | | 78 | | | |

Table S1: Thin film morphology (film thickness and mass density converted from the average electron density) of films in the thickness range from 1.5 nm to 12 nm as determined by fits of the X-ray reflectivity curves. The given mean squared errors are given on logarithmic scale of the data. Vertical size of the crystallites obtained from the Bragg peak at $q_z = 2.38$ nm$^{-1}$.
